# Submaxillary Calculus

**Published:** 1887-05

**Authors:** 


### Submaxillary Calculus.

Dr. Collis Barry thus writes in the *Lancet:*

The following case is worthy of record on account of the unusual size of the calculus and the gravity of the symptoms resulting from its presence.

R. W., aged sixty-six, consulted me early in September last concerning a swelling beneath the lower jaw on the right side, which caused him extreme difficulty in swallowing. Flashing pains came on at intervals, and extended radially from the swelling upwards into the face and head, inwards towards the tongue, and downwarde as far as the right shoulder and arm. These symptoms had existed for about eight months, becoming gradually more severe; latterly the patient had begun to lose flesh, and his friends became alarmed at his condition.

Upon examination I found a hard substance corresponding in position with the submaxillary gland placed just below the ramus of the inferior maxilla, rounded in form, and more easily distinguishable from the outer side than from the interior of the mouth, and about the size of a small walnut. By pressure from the outside, a few drops purulent discharge appeared at the orifice of Wharton's duct. There was no hardness or concretion at the mouth of the duct, as usually met with in cases of salivary calculus, and there was no induration of the cervical glands. Taking into consideration the gravity of the symptoms and the increasing loss of strength, I suggested early removal, but requested a consultation. Dr. Ainsley, of Hartlepool, saw the case with me, and confirmed my suggestion. On October 12th, chloroform being administered by Dr. Ainsley, I proceeded to cut down upon the submaxillary gland by a vertical incision about a quarter of an inch anterior to the facial artery as it crosses the inferior maxilla, extending downwards about two inches; a second cross incision was made to make room. There was considerable hemorrhage from the submaxillary and other small branches of the facial. The whole of the gland superficial to the mylo-hyoid was removed, and the calculus could then be distinctly felt at the commencement of the duct, and apparently imbedded in the gland. A few of the posterior fibres of the mylo-hyoid were divided, and the calculus seized with vulsellum forceps and removed. Upon examination after removal, the calculus proved to be phosphatic, and measured one inch by half an inch approximately (the exact measurement in length being uncertain, owing to the crumbling of a portion of the calculus beneath the vulsellum forceps). The wound was plugged with lint soaked in turpentine; eucalyptus ointment and iodoform being substituted on the following day. The subsequent history of the case was one of uninterrupted recovery, the wound granulating freely and becoming entirely healed in three weeks from the date of operation, all the symptoms previously complained of having entirely disappeared.

*Remarks.*—The ordinary method of removal by incision of the mucous membrane was impracticable in this case, owing to the depth of the calculus from the surface of the mucous membrane. Erichsen mentions, as the largest calculus removed, one of the size of a small damson stone; but, so far as I can ascertain, no mention has been made of a calculus of this size occupying so deep a position.

